# The forgotten bullet: a rare coronary-CT image discovery

**DOI:** 10.1007/s10554-025-03374-1

**Published:** 2025-03-24

**Authors:** Philippe Musso, Anne-Lise Hachulla, Christoph Gräni

**Affiliations:** 1 Department of Cardiology, Bern University Hospital, Inselspital, University of Bern, Bern, Switzerland; 2 Centre médical du Lac, Route Suisse 35, Gland, 1196 Switzerland; 3 Clinique de Genolier, Route du Muids 3, Genolier, 1272 Switzerland

**Keywords:** Multimodality imaging, CCTA, Cardiac computed tomography, Bullet, Foreign body

*Category “Image” The International Journal of Cardiovascular Imaging*.

A 67-year-old male with no history of cardiac disease or cardiovascular interventions presented to the cardiologist with atypical chest pain. A coronary computed tomography angiography could rule out stenotic coronary artery disease. However, an unclear hyper-attenuated structure was visible on both the native imaging (Panel A, red arrow) and the contrast-enhanced imaging (Panel B, C) in the mediastinum, close to the aorta and the pulmonary artery. At this stage, in a patient without a known medical history, possible causes for such a structure included various implanted medical devices, such as a patent foramen ovale occluder, an embolized left atrial appendage occluder, an edge-to-edge clip device, or any other embolized metal, cement, or calcification (scout imaging Panel D, E). Upon further questioning, the patient recalled a traumatic incident that had occurred seventeen years earlier. At the time, he was working as a local milk vendor in Colombia and had been involved in a shooting incident. One bullet entered through his nose, traveled through his right eye, and eventually lodged in his right ear. Another bullet first perforated the liver and intestines before ending up in the mediastinum, positioned between the right posterior and lateral surface of the ascending aorta and the anterior surface of the superior vena cava (3D reconstruction, Panel F, G) with indentation on the right pulmonary artery and the left atrium. At that time, the surgeons decided not to remove the bullets as the patient was stable, and after twelve days of hospitalization, the patient was discharged. As a result of his injuries, he is now blind in his right eye and deaf in his right ear. Since coronary artery disease could be excluded and the patient only recently developed atypical chest pain, the bullet was not considered the source of his symptoms but rather an incidental imaging finding. Retained bullets within the mediastinum / around heart are extremely rare. This case highlights the need for a detailed patient history, particularly in individuals undergoing cardiac imaging, where, besides coronary computed tomography, modalities such as stress cardiac magnetic resonance imaging are also potential options for evaluating suspected coronary artery disease. Importantly, in this patient, any magnetic resonance imaging scan is strictly contraindicated due to the presence of retained bullets in both close to the heart and the head.

**Figure:**
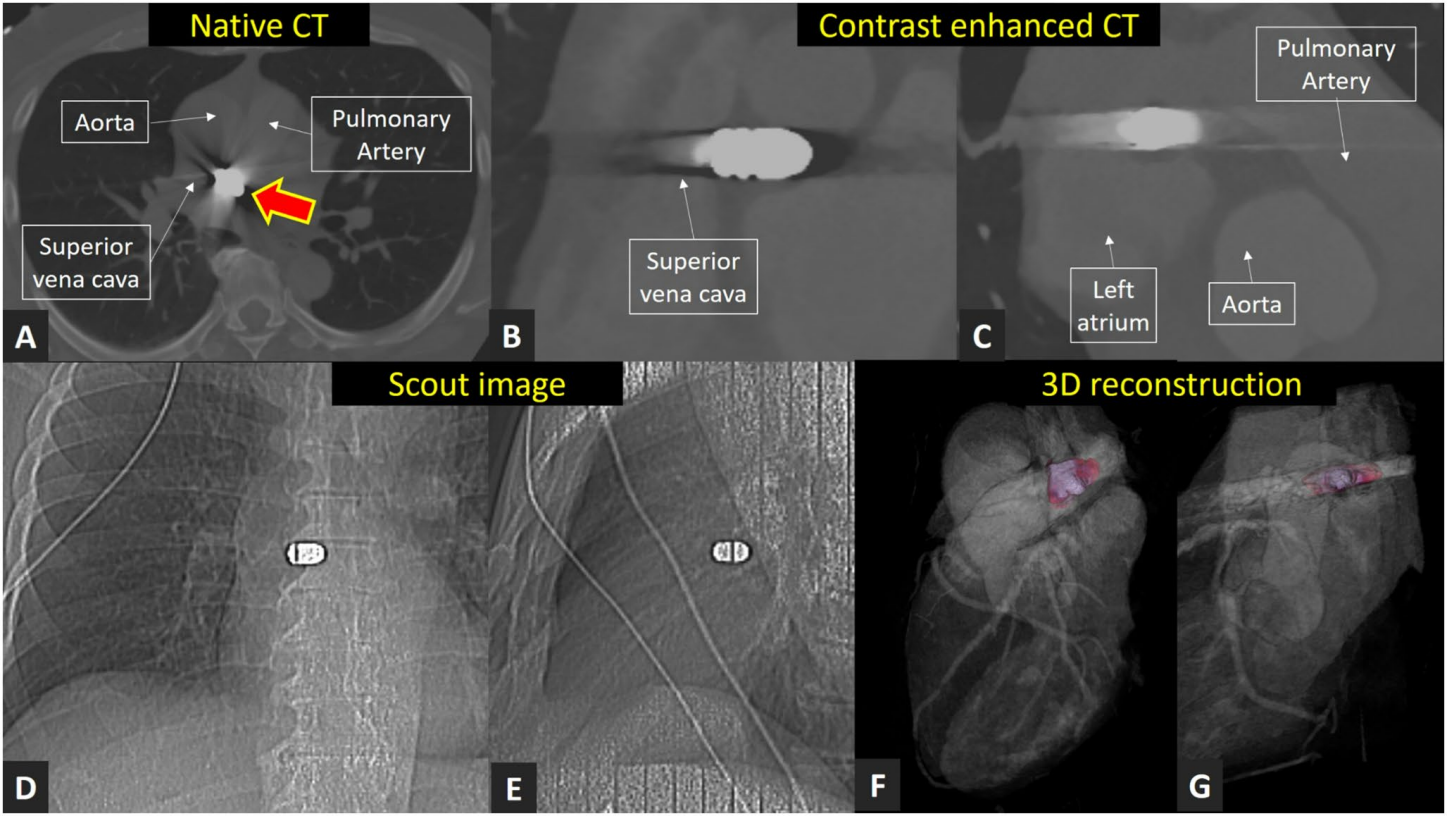

## Data Availability

No datasets were generated or analysed during the current study.

